# Fluorescein in brain metastasis and glioma surgery

**DOI:** 10.1007/s00701-015-2576-4

**Published:** 2015-09-18

**Authors:** Walter Stummer

**Affiliations:** Department of Neurosurgery, University of Münster, Albert-Schweitzer-Campus 1, Gebäude A1, 48149 Münster, Germany

Dear Sir,

I appreciate the opportunity for commenting on a Letter by Acerbi and coworkers concerning our past submission (http://link.springer.com/article/10.1007%2Fs00701-015-2471-z).

Fluorescein is being investigated for fluorescence-guided resections of brain metastasis and gliomas [1–3].

Eager to exploit the new potential of this application, our group is also exploring fluorescein, and has been exploring it for a while. However, in our hands we are having continuing problems using this method and are simply not observing the selectivity that we would desire.

In our experience with metastases (Fig. 1) and malignant gliomas (Supplementary Video), significant fluorescein fluorescence is found after resection at the margins in obviously normal and perifocal edematous brain tissue despite all efforts to follow the guidance of Dr. Acerbi and coworkers using low doses (4 mg/kg) given with induction of anesthesia and performing surgery with the Zeiss Yellow 560 filter system. This confounds applicability.

**Fig. 1:**
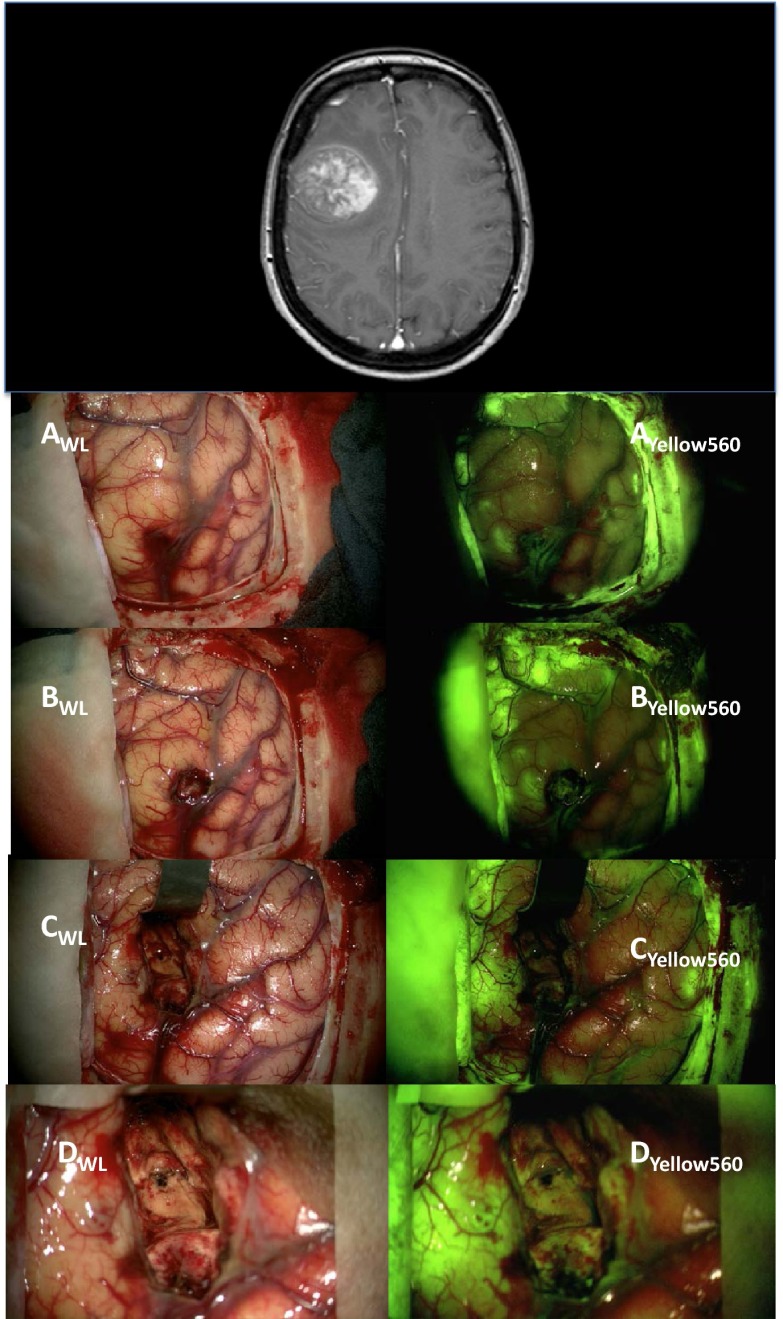
Metastasis of adenocarcinoma with perifocal edema, operated on using fluorescein (4 mg/kg i.v., injected immediately after induction of anesthesia; Zeiss Yellow 560, filter) as previously described [1, 2]. **a** Before corticotomy; note reduced cortical fluorescence covering the tumor, indicating reduced perfusion by fluorescein. Much more fluorescence is found cortically away from the tumor, where perfusion is greater and edema reaches the cortical surface (image captured 72 min after injection). **b** After opening the cortex; little fluorescence is found in the tumor. In the meantime, more fluorescein is accumulating in perifocal cortical tissue (image obtained 96 min after injection and frozen section confirming metastasis). **c** After resection of tumor. The entire cavity fluoresces. There is unspecific fluorescence in the adjacent (edematous) cortex (image obtained 144 min after injection). **d** Higher magnification of image **c** (*WL* white light illumination; *Yellow560* utilizing the Zeiss Yellow 560 filter)

We find this worrisome. Our differing vantage points have obviously now generated a very lively discussion. Dr. Acerbi et al. feel (wrongly) accused, with accusations certainly not having been my intention. I highly respect their efforts to improve the surgical management of brain tumor patients. Nevertheless, I do critically reiterate that I believe the use of fluorescein outside of studies to be premature and requiring further investigation in a complex field in which histology, timing, dose, illumination, tissue perfusion, and edema play a role, with a marker of blood–brain barrier integrity that offers a tissue signal that is not simply binary. Others are critical as well [4, 5]. Dr. Acerbi et al. are thankfully involved in such studies and others and I are awaiting their results.

Sincerely,

Walter Stummer

## Electronic supplementary material

Video Glioblastoma surgery using fluorescein (4 mg/kg, given with induction of anesthesia, Zeiss Yellow 560 filter) after tumor debulking. Again, there is unclear fluorescence at the resection margins and in the cortex which is outside the region defined as tumor on the MRI. (M4V 57600 kb)
